# The BioMart community portal: an innovative alternative to large, centralized data repositories

**DOI:** 10.1093/nar/gkv350

**Published:** 2015-04-20

**Authors:** Damian Smedley, Syed Haider, Steffen Durinck, Luca Pandini, Paolo Provero, James Allen, Olivier Arnaiz, Mohammad Hamza Awedh, Richard Baldock, Giulia Barbiera, Philippe Bardou, Tim Beck, Andrew Blake, Merideth Bonierbale, Anthony J. Brookes, Gabriele Bucci, Iwan Buetti, Sarah Burge, Cédric Cabau, Joseph W. Carlson, Claude Chelala, Charalambos Chrysostomou, Davide Cittaro, Olivier Collin, Raul Cordova, Rosalind J. Cutts, Erik Dassi, Alex Di Genova, Anis Djari, Anthony Esposito, Heather Estrella, Eduardo Eyras, Julio Fernandez-Banet, Simon Forbes, Robert C. Free, Takatomo Fujisawa, Emanuela Gadaleta, Jose M. Garcia-Manteiga, David Goodstein, Kristian Gray, José Afonso Guerra-Assunção, Bernard Haggarty, Dong-Jin Han, Byung Woo Han, Todd Harris, Jayson Harshbarger, Robert K. Hastings, Richard D. Hayes, Claire Hoede, Shen Hu, Zhi-Liang Hu, Lucie Hutchins, Zhengyan Kan, Hideya Kawaji, Aminah Keliet, Arnaud Kerhornou, Sunghoon Kim, Rhoda Kinsella, Christophe Klopp, Lei Kong, Daniel Lawson, Dejan Lazarevic, Ji-Hyun Lee, Thomas Letellier, Chuan-Yun Li, Pietro Lio, Chu-Jun Liu, Jie Luo, Alejandro Maass, Jerome Mariette, Thomas Maurel, Stefania Merella, Azza Mostafa Mohamed, Francois Moreews, Ibounyamine Nabihoudine, Nelson Ndegwa, Céline Noirot, Cristian Perez-Llamas, Michael Primig, Alessandro Quattrone, Hadi Quesneville, Davide Rambaldi, James Reecy, Michela Riba, Steven Rosanoff, Amna Ali Saddiq, Elisa Salas, Olivier Sallou, Rebecca Shepherd, Reinhard Simon, Linda Sperling, William Spooner, Daniel M. Staines, Delphine Steinbach, Kevin Stone, Elia Stupka, Jon W. Teague, Abu Z. Dayem Ullah, Jun Wang, Doreen Ware, Marie Wong-Erasmus, Ken Youens-Clark, Amonida Zadissa, Shi-Jian Zhang, Arek Kasprzyk

**Affiliations:** 1 Wellcome Trust Sanger Institute, Welcome Trust Genome Campus, Hinxton, CB10 1SD, UK; 2 The Weatherall Institute Of Molecular Medicine, University of Oxford, Oxford, OX3 9DS, UK; 3 Genentech, Inc. 1 DNA Way South San Francisco, CA 94080, USA; 4 Center for Translational Genomics and Bioinformatics San Raffaele Scientific Institute, Via Olgettina 58, 20132 Milan, Italy; 5 Dept of Molecular Biotechnology and Health Sciences University of Turin, Italy; 6 European Molecular Biology Laboratory, European Bioinformatics Institute, Wellcome Trust Genome Campus, Hinxton, Cambridge, CB10 1SD, UK; 7 Institute for Integrative Biology of the Cell (I2BC), CEA, CNRS, Université Paris Sud, 1 avenue de la terrasse, 91198 Gif sur Yvette, France; 8 Department of Electrical and Computer Engineering, Faculty of Engineering, King Abdulaziz University, Jeddah, Saudi Arabia; 9 MRC Human Genetics Unit, Institute of Genetics and Molecular Medicine, Western General Hospital, Edinburgh, EH4 2XU, UK; 10 Sigenae, INRA, Castanet-Tolosan, France; 11 Department of Genetics, University of Leicester, University Road, Leicester, LE1 7RH, UK; 12 MRC Harwell, Harwell Science and Innovation Campus, Oxfordshire, OX11 0RD, UK; 13 International Potato Center (CIP), Lima, 1558, Peru; 14 Department of Energy, Joint Genome Institute, Walnut Creek, USA; 15 Centre for Molecular Oncology, Barts Cancer Institute, Queen Mary University of London, Charterhouse Square, London EC1M 6BQ, UK; 16 IRISA-INRIA, Campus de Beaulieu 35042 Rennes, France; 17 Laboratory of Translational Genomics, Centre for Integrative Biology, University of Trento, Trento, Italy; 18 Center for Mathematical Modeling and Center for Genome Regulation, University of Chile, Beauchef 851, 7th floor, Chile; 19 Plate-forme bio-informatique Genotoul, Mathématiques et Informatique Appliquées de Toulouse, INRA, Castanet-Tolosan, France; 20 Oncology Computational Biology, Pfizer, La Jolla, USA; 21 Catalan Institute for Research and Advanced Studies (ICREA), Passeig Lluis Companys 23, E-08010 Barcelona, Spain; 22 Universitat Pompeu Fabra, Dr Aiguader 88 E-08003 Barcelona, Spain; 23 Kasuza DNA Research Institute, Chiba, 292–0818, Japan; 24 HUGO Gene Nomenclature Committee (HGNC), European Bioinformatics Institute (EMBL-EBI) Wellcome Trust Genome Campus, Hinxton, CB10 1SD, UK; 25 Medicinal Bioconvergence Research Center, College of Pharmacy, Seoul National University, Seoul 151–742, Republic of Korea; 26 Department of Molecular Medicine and Biopharmaceutical Sciences, Seoul National University, Seoul 151–742, Republic of Korea; 27 Research Institute of Pharmaceutical Sciences, College of Pharmacy, Seoul National University, Seoul 151–742, Republic of Korea; 28 Information Center for Bio-pharmacological Network, Seoul National University, Suwon 443–270, Republic of Korea; 29 Ontario Institute for Cancer Research, Toronto, M5G 0A3, Canada; 30 RIKEN Center for Life Science Technologies (CLST), Division of Genomic Technologies (DGT), Kanagawa, 230–0045, Japan; 31 School of Dentistry and Dental Research Institute, University of California Los Angeles (UCLA), Los Angeles, CA 90095–1668, USA; 32 Iowa State Univeristy, USA; 33 Mouse Genomic Informatics Group, The Jackson Laboratory, Bar Harbor, ME 04609, USA; 34 RIKEN Preventive Medicine and Diagnosis Innovation Program, Saitama 351–0198, Japan; 35 INRA URGI Centre de Versailles, bâtiment 18 Route de Saint Cyr 78026 Versailles, France; 36 Center for Bioinformatics, State Key Laboratory of Protein and Plant Gene Research, College of Life Sciences, Peking University, Beijing, 100871, P.R. China; 37 VectorBase, European Bioinformatics Institute, Wellcome Trust Genome Campus, Hinxton, CB10 1SD, UK; 38 Institute of Molecular Medicine, Peking University, Beijing, China; 39 Computer Laboratory, University of Cambridge, Cambridge, CB3 0FD, UK; 40 Department of Mathematical Engineering, University of Chile, Av. Beauchef 851, 5th floor, Santiago, Chile; 41 Departament of Biochemistry, Faculty of Science for Girls, King Abdulaziz University, Jeddah, Saudi Arabia; 42 Department of Medical Epidemiology and Biostatistics, Karolinska Institutet, PO Box 281, 17177 Stockholm, Sweden; 43 Inserm U1085 IRSET, University of Rennes 1, 35042 Rennes, France; 44 Department of Biological Sciences, Faculty of Science for Girls, King Abdulaziz University, Jeddah, Saudi Arabia; 45 Cold Spring Harbor Laboratory, Cold Spring Harbor, NY 11724, USA; 46 Eagle Genomics Ltd., Babraham Research Campus, Cambridge, CB22 3AT, UK; 47 Human Longevity, Inc. 10835 Road to the Cure 140 San Diego, CA 92121, USA; 48 Department of Biological Sciences, Faculty of Science, King Abdulaziz University, Jeddah, Saudi Arabia

## Abstract

The BioMart Community Portal (www.biomart.org) is a community-driven effort to provide a unified interface to biomedical databases that are distributed worldwide. The portal provides access to numerous database projects supported by 30 scientific organizations. It includes over 800 different biological datasets spanning genomics, proteomics, model organisms, cancer data, ontology information and more. All resources available through the portal are independently administered and funded by their host organizations. The BioMart data federation technology provides a unified interface to all the available data. The latest version of the portal comes with many new databases that have been created by our ever-growing community. It also comes with better support and extensibility for data analysis and visualization tools. A new addition to our toolbox, the enrichment analysis tool is now accessible through graphical and web service interface. The BioMart community portal averages over one million requests per day. Building on this level of service and the wealth of information that has become available, the BioMart Community Portal has introduced a new, more scalable and cheaper alternative to the large data stores maintained by specialized organizations.

## INTRODUCTION

The methods of data generation and processing that are utilized in biomedical sciences have radically changed in recent years. With the advancement of new high-throughput technologies, data have grown in terms of quantity as well as complexity. However, the significance of the information that is hidden in the newly generated experimental data can only be deciphered by linking it to other types of biological data that have been accumulated previously. As a result there are already numerous bioinformatics resources and new ones are constantly being created. Typically, each resource comes with its own query interface. This poses a problem for the scientists who want to utilize such resources in their research. Even the simplest task such as compiling results from a few existing resources is challenging due to the lack of a complete, up to date catalogue of already existing resources and the necessity of constantly learning how to navigate new query interfaces. A different challenge is faced by collaborating groups of scientists who independently generate or maintain their own data. Such collaborations are seriously hampered by the lack of a simple data management solution that would make it possible to connect their disparate, geographically distributed data sources and present them in a uniform way to other scientists. The BioMart project has been set up to address these challenges.

## SOFTWARE

BioMart is an open source data management system, which is based on a data federation model (1). Under this model, each data source is managed, updated and released independently by their host organization while the BioMart software provides a unified view of these sources that are distributed worldwide. The data sources are presented to the user through a unified set of graphical and programmatic interfaces so that they appear to be a single integrated database. To navigate this database and compile a query the user does not have to learn the underlying structure of each data source but instead use a set of simple abstractions: datasets, filters and attributes. Once a user's input is provided, the software distributes parts of the query to individual data sources, collects the data and presents the user with the unified result set.

The BioMart software is data agnostic and its applications are not limited to biological data. It is cross-platform and supports many popular relational database managements systems, including MySQL, Oracle, PostgreSQL. It also supports many third party packages such as Taverna (2), Galaxy (3), Cytoscape (4) and biomaRt (5), which part of the Bioconductor (6) library.

The BioMart project currently maintains two independent code bases: one written in Java and one written in Perl. For more information about the architecture and capabilities of each of the packages please refer to previous publications (1,7). The latest version of the Java based BioMart software has been significantly enhanced with new additions to the existing collection of graphical user interfaces (GUIs). It has also been re-engineered to provide better support and extensibility for data analysis and visualization tools. The first of the BioMart tools based on this new framework has already been implemented and is accessible from the BioMart Community Portal.

The BioMart project adheres to the open source philosophy that promotes collaboration and code reuse. Two good examples of how this philosophy benefits the scientific community are provided by two independent research groups. The INRA group based in Toulouse, France has recently released a software package called RNAbrowse (RNA-Seq De Novo Assembly Results Browser) (8). The Pfizer group based in La Jolla, USA has just announced the release of OASIS: A Web-based Platform for Exploratory Analysis of Cancer Genome and Transcriptome data (www.oasis-genomics.org). Both of these software packages are based on the BioMart software.

## DATA

**Figure 1.**
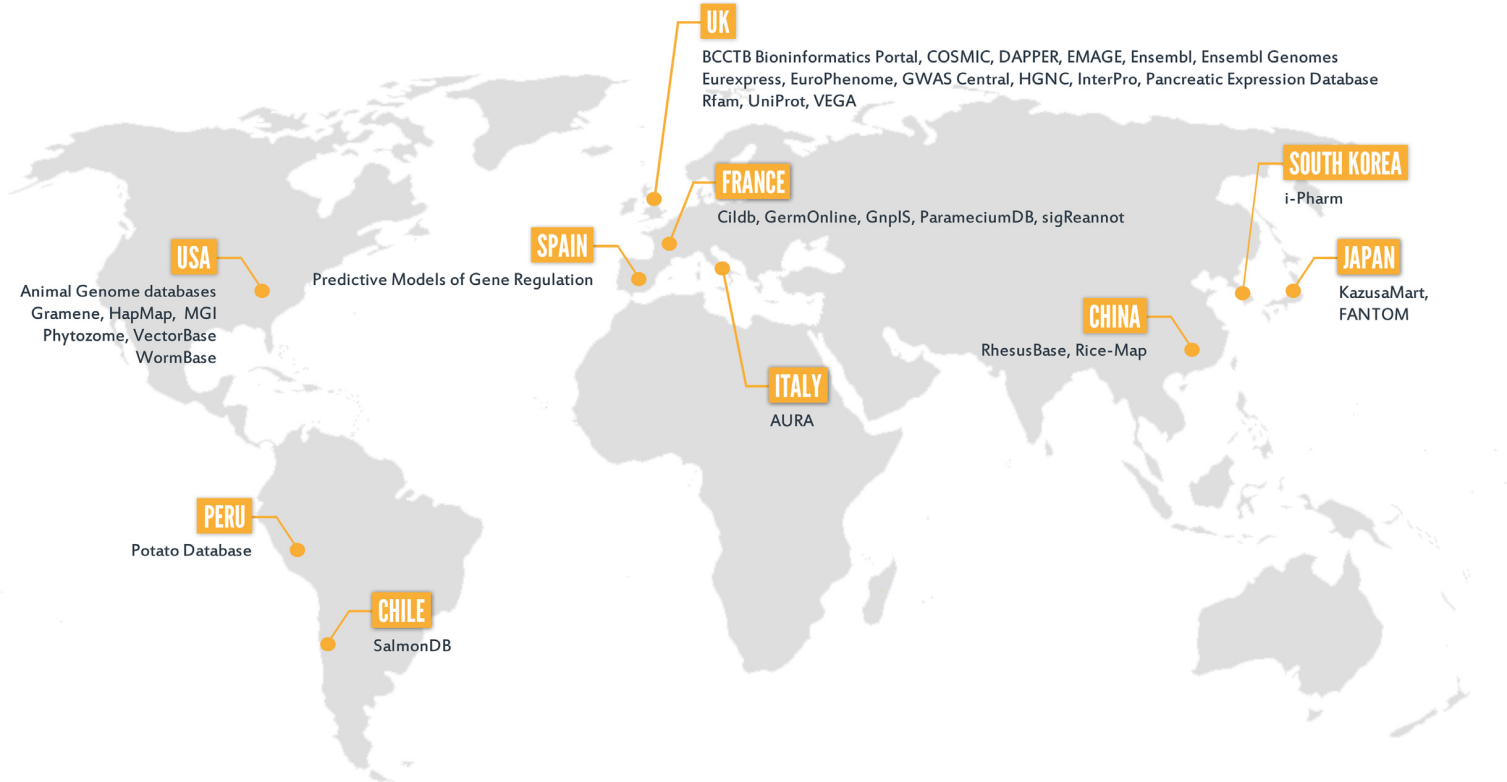
BioMart community databases and their host countries.

The BioMart community consists of a wide spectrum of different research groups that use the BioMart technology to provide access to their databases. It currently comprises 30 scientific organizations supporting 38 database projects that contain over 800 different biological datasets spanning genomics, proteomics, model organisms, cancer data, ontology information and more. The BioMart community is constantly growing and since the last publication (9), 11 new database projects have become available. As new BioMart databases become available locally they also become gradually integrated into the BioMart Community Portal. The main function of the portal is to provide a convenient single point of access to all available data that is distributed worldwide (Figure 1). All BioMart databases that are included in the portal are independently administered and funded. Table 1 provides a detailed list of all BioMart community resources as of March 2015.

**Table 1.** BioMart community databases and their host organizations

Database	Description	Host	Reference
Animal Genome databases^a,b^	Agriculturally important livestock genomes	Iowa State University, US	NA
Atlas of UTR Regulatory Activity (AURA)^a^	Meta-database centred on mapping post-transcriptional (PTR) interactions of trans-factors with human and mouse untranslated regions (UTRs) of mRNAs	University of Trento, Italy	(36)
BCCTB Bioinformatics Portal^a^	Portal for mining omics data on breast cancer from published literature and experimental datasets	Breast Cancer Campaign/Barts Cancer Institute UK	(37)
Cildb	Database for eukaryotic cilia and centriolar structures, integrating orthology relationships for 44 species with high-throughput studies and OMIM	Centre National de la Recherche Scientifique (CNRS), France	(38)
COSMIC	Somatic mutation information relating to human cancers	Wellcome Trust Sanger Institute (WTSI), UK	(39)
DAPPER^a^	Mass spec identified protein interaction networks in *Drosophila* cell cycle regulation	Department of Genetics, University of Cambridge, Cambridge, UK	NA
EMAGE	In situ gene expression data in the mouse embryo	Medical Research Council, Human Genetics Unit (MRC HGU), UK	(40)
Ensembl	Genome databases for vertebrates and other eukaryotic species	Wellcome Trust Sanger Institute (WTSI), UK	(41)
Ensembl Genomes	Ensembl Fungi, Metazoa, Plants and Protists	European Bioinformatics Institute (EBI), UK	(41)
Euraexpress	Transcriptome atlas database for mouse embryo	Medical Research Council, Human Genetics Unit (MRC HGU), UK	(42)
EuroPhenome	Mouse phenotyping data	Harwell Science and Innovation Campus (MRC Harwell), UK	(15)
FANTOM5^a^	The FANTOM5 project mapped a promoter level expression atlas in human and mouse. The FANTOM5 BioMart instance provides the set of promoters along with annotation.	RIKEN Center for Life Science Technologies (CLST), Japan	(16)
GermOnLine	Cross-species microarray expression database focusing on germline development, meiosis, and gametogenesis as well as the mitotic cell cycle	Institut national de la santé et de la recherche médicale (Inserm), France	(17)
GnpIS^a^	Genetic and Genomic Information System (GnpIS)	Institut Nationale de Recherche Agronomique (INRA), Unité de Recherche en Génomique-Info (URGI), France	(18)
Gramene	Agriculturally important grass genomes	Cold Spring Harbor Laboratory (CSHL), US	(43)
GWAS Central^a^	GWAS Central provides a comprehensive curated collection of summary level findings from genetic association studies	University of Leicester, UK	(19)
HapMap	Multi-country effort to identify and catalog genetic similarities and differences in human beings	National Center for Biotechnology Information (NCBI), US	(20)
HGNC	Repository of human gene nomenclature and associated resources	European Bioinformatics Institute (EBI), UK	(21)
i-Pharm^a^	PharmDB-K is an integrated bio-pharmacological network databases for TKM (Traditional Korean Medicine)	Information Center for Bio-pharmacological Network (i-Pharm), South Korea	(22)
InterPro	Integrated database of predictive protein ‘signatures’ used for the classification and automatic annotation of proteins and genomes	European Bioinformatics Institute (EBI), UK	(44)
KazusaMart	Cyanobase, rhizobia, and plant genome databases	Kazusa DNA Research Institute (Kazusa), Japan	NA
MGI	Mouse genome features, locations, alleles, and orthologs	Jackson Laboratory, US	(23)
Pancreatic Expression Database	Results from published literature	Barts Cancer Institute UK	(24)
ParameciumDB	Paramecium genome database	Centre National de la Recherche Scientifique (CNRS), France	(25)
Phytozome	Comparative genomics of green plants	Joint Genome Institute (JGI)/Center for Integrative Genomics (CIG), US	(26)
Potato Database	Potato and sweetpotato phenotypic and genomic information	International Potato Center (CIP), Peru	NA
PRIDE	Repository for protein and peptide identifications	European Bioinformatics Institute (EBI), UK	(45)
Regulatory Genomics Group^a^	Predictive Models of Gene Regulation from High-Throughput Epigenomics Data	Universitat Pompeu Fabra (UPF), Spain	(27)
Rfam^a^	The Rfam database is a collection of RNA families, each represented by multiple sequence alignments, consensus secondary structures and covariance models (CMs).	Wellcome Trust Sanger Institute (WTSI), UK	(28)
RhesusBase^a^	A knowledgebase for the monkey research community	Peking University, China	(29)
Rice-Map	Rice (japonica and indica) genome annotation database	Peking University, China	(30)
SalmonDB	Genomic information for Atlantic salmon, rainbow trout, and related species	Center for Mathematical Modeling and Center for Genome Regulation (CMM), Chile	(31)
sigReannot	Aquaculture and farm animal species microarray probes re-annotation	INRA - French National Institute of Agricultural Research, France	(46)
UniProt	Protein sequence and functional information	European Bioinformatics Institute (EBI), UK	(32)
VectorBase	Genome information for invertebrate vectors of human pathogens	University of Notre Dame, US	(33)
VEGA	Manual annotation of vertebrate genome sequences	Wellcome Trust Sanger Institute (WTSI), UK	(34)
WormBase	*C. elegans* and related nematode genomic information	Cold Spring Harbor Laboratory (CSHL), US	(35)

^a^Denotes new databases that have become available since last publication (9).

^b^Denotes new databases that are not yet integrated into the portal.

## PORTAL

**Figure 2.**
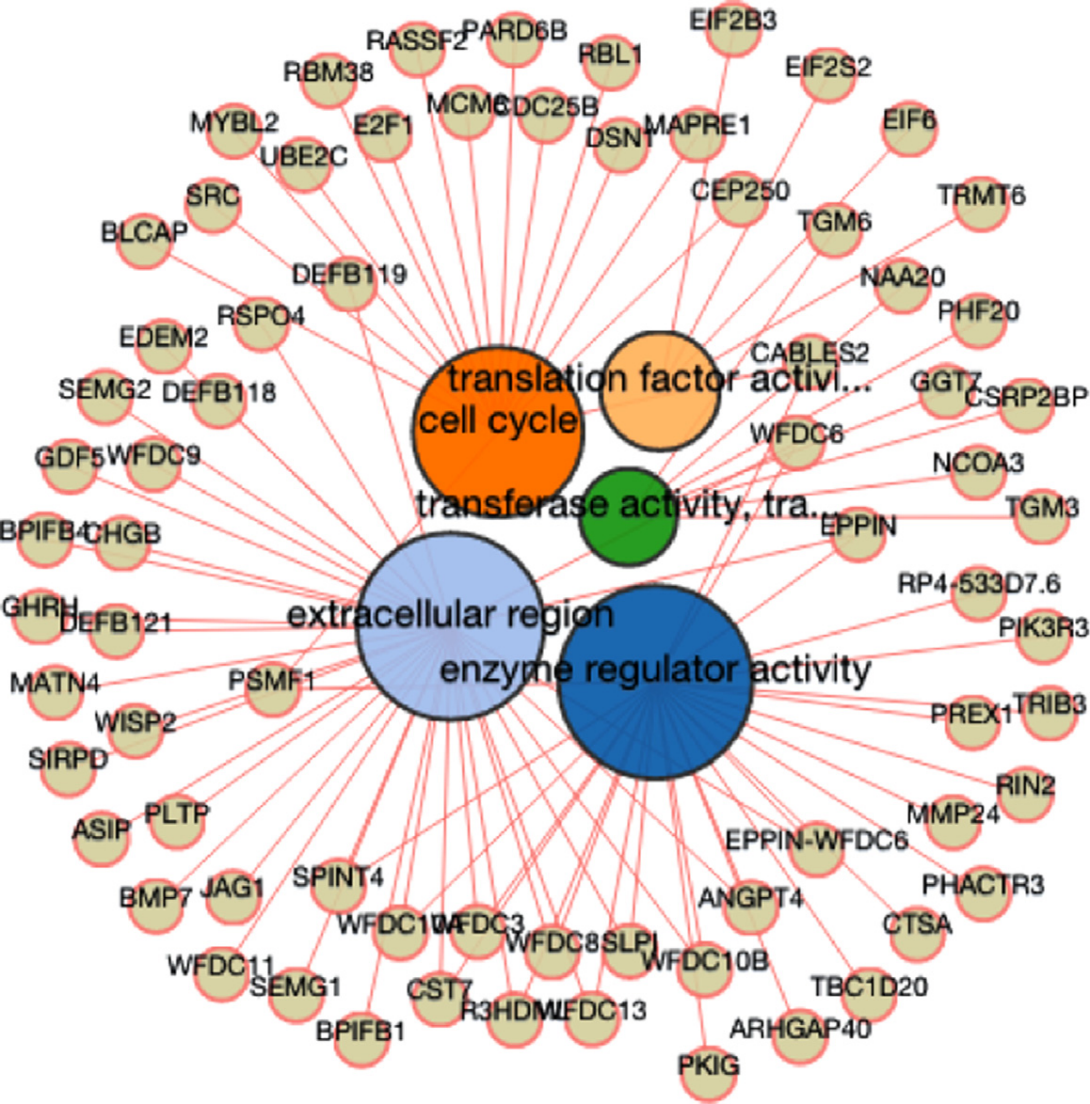
The network graphic output of the BioMart enrichment tool. The Gene Ontology (GO) enrichment analysis was performed using BED file containing human data. This tool is also accessible through web services (Java version only). The programmatic access complies with a standard BioMart interface: dataset, filter and attribute.

The current version of the BioMart Community Portal operates two different instances of the web server: one implemented in Perl and the other in Java. Both servers support complex database searches and although they use different types of GUIs, they share the same navigation and query compilation logic based on selection of datasets, filters and attributes (9,10). The Java version of the portal also includes a section for specialized tools, which consists of the following: Sequence retrieval, ID Converter and Enrichment Analysis. Sequence retrieval allows easy querying of sequences while the ID Converter tool allows users to enter or upload a list of identifiers in any format (currently supported by Ensembl), and retrieve the same list converted to any other supported format. The enrichment tool supports enrichment analysis of genes in all species included in the current Ensembl release. For each of those species a broad range of gene identifiers is available. Furthermore, the tool supports cross species analysis using Ensembl homology data. For instance, it is possible to perform a one step enrichment analysis against a human disease dataset using experimental data from any of the species for which human homology data is available. Finally, the enrichment tool facilitates analysis of BED files containing genomic features such as Copy Number Variations or Differentially Methylated Regions. The output is provided in tabular and network graphic format (Figure 2).

## WEB SERVICE

The BioMart Community Portal handles queries from several interfaces such as:

- PERL API
- Java API
- Web interfaces
- URL based access
- RESTful web service
- SPARQL

**Figure 3.**
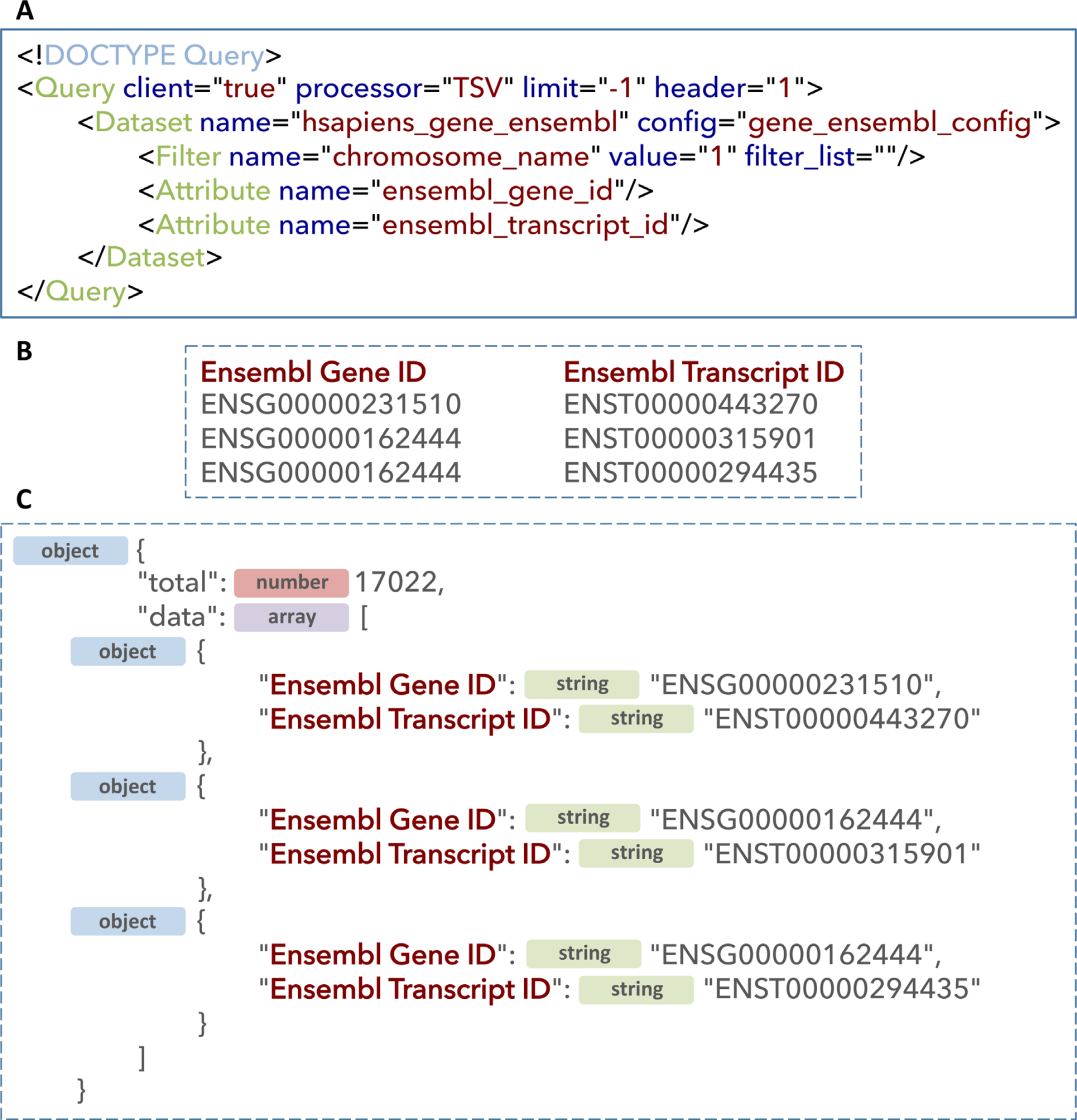
The XML web service query (**A**) and the corresponding two types of output: tab delimited following setting a processor to ‘TSV’ (**B**) and JSON following setting processor to ‘JSON’.

For more detailed description of all the interfaces please refer to earlier publications (1,7). In the section below we provide a description and compare the REST-based web service, which is implemented in Perl and its counterpart, which is implemented in Java. It is worth noting that the web service maintains the same query interface both in Perl and Java implementations. For example, the web service query (Figure 3A) can be run against java-based server as follows:

curl –data-urlencode query@query.xml http://central.biomart.org/martservice/results
or its Perl-based counter-part as below
curl –data-urlencode query@query.xml http://www.biomart.org/biomart/martservice

By default, query sets the attribute processor to ‘TSV’ requesting tab-delimited results (Figure 3B). Alternatively, by setting processor to ‘JSON’, would return JSON formatted results (Figure 3C), which are readily consumable by third-party web-based clients saving overhead of parsing and format translations. Please note that JSON format is only available in the java version.

A simple way to compile a web service query for later programmatic use is to use one of the web GUIs and generate the query XML using REST/SOAP button. After following the steps outlined by the GUI and clicking the ‘results’ button, the user needs to click the REST/SOAP button, save the query and run it as described above. Alternatively a user can take advantage of the programmatic access to all the metadata defining marts, datasets, filters and attributes. The access to the metadata served by the Java and Perl BioMart servers is provided using the following webservice requests:

Java (central.biomart.org)

- registry information:
  http://central.biomart.org/martservice/portal
- available marts:
  http://central.biomart.org/martservice/marts
- datasets available for a config:
  http://central.biomart.org/martservice/datasets?config=snp_config
- attributes available for a dataset:
  http://central.biomart.org/martservice/attributes?datasets=btaurus_snp&config=snp_config
- filters available for a dataset:
  http://central.biomart.org/martservice/filters?datasets=btaurus_snp&config=snp_config

Perl (www.biomart.org)

- registry information:
  http://www.biomart.org/biomart/martservice?type=registry
- datasets available for a mart:
  http://www.biomart.org/biomart/martservice?type=datasets&mart=ensembl
- attributes available for a dataset:
  http://www.biomart.org/biomart/martservice?type=attributes&dataset=oanatinus_gene_ensembl
- filters available for a dataset:
  http://www.biomart.org/biomart/martservice?type=filters&dataset=oanatinus_gene_ensembl
- configuration for a dataset:
  http://www.biomart.org/biomart/martservice?type=configuration&dataset=oanatinus_gene_ensembl

Please note that the granularity between mart and dataset has been improved in the Java version through the introduction of multiple dataset configs. This facilitates the end-users to browse various views of the same dataset, which are presented through the portal either using a different GUI or subsets of data.

## QUERY EXAMPLES

Given the coverage of the current BioMart datatsets, many relevant biological questions can be answered. For example, a researcher who has detected potentially pathogenic variants in FGFR2 (ENSG00000066468) from exome sequencing patients may be interested if the same variants have been previously described and if they were associated with the same or similar diseases. To answer this, integrated data from Ensembl can be queried as shown in Table 2 to display all known variants annotated within FGFR2 that are predicted as pathogenic by SIFT (11) and Polyphen (12). The genomic position outputs can be compared to the researcher's variants and the phenotype data used to assess candidacy for their cases. For example, the first batch of results shows a C->G variant at position 121520160 on chromosome 10 that is associated with Apert syndrome (OMIM:176943).

**Table 2.** Query to display phenotypic consequence for known, pathogenic variants in FGFR2

Database and dataset	Filters	Attributes
Ensembl 78 Short Variations	Ensembl Gene ID(s):	Chromosome name
(WTSI, UK)	ENSG00000066468	Chromosome position start (bp)
Homo sapiens Short Variation (SNPs and indels) (GRCh38)	SIFT Prediction: deleterious	Chromosome position end (bp)
	PolyPhen Prediction: probably damaging	Strand
		Variant Alleles
		Ensembl Gene ID
		Consequence to transcript
		Associated variation names
		Study External Reference
		Source name
		Associated gene with phenotype
		Phenotype description

Another common use case that BioMart is used for is to analyse a list of genes to establish whether they are associated with particular protein functions, pathways or diseases more often than would be expected by chance (enrichment analysis). For example, a researcher may have discovered that AURKA, AURKB, AURKC, PLK1, CDK1 and CDK4 are differentially expressed in their experiment and used BioMart's enrichment tool with its default settings to analyse these genes. The results show that these genes are enriched for involvement in the cell cycle, kinase activity and mitotic nuclear division amongst others. Many other real usage examples are documented in our previous paper (10) and the BioMart special issue in Database: the journal of biological databases and biocuration (www.oxfordjournals.org/our_journals/databa/biomart_virtual_issue.html).

## CONCLUSIONS

Since its conception as a data-mining interface for the Human Genome Project (13) BioMart has rapidly grown to become an international collaboration involving a large number of different groups and organizations both in academia and in industry (14). It has been successfully applied to many different types of data including genomics, proteomics, model organisms, cancer data, etc., proving that its generic data model is widely applicable (15–53). BioMart has also provided a first successful solution for the unprecedented data management needs of the International Cancer Genome Consortium proving that the federated model scales well with the amounts of data generated by Next Generation Sequencing (48).

There are a number of important factors that contributed to the BioMart's success and its adoption by many different types of projects around the world as their data management platform. BioMart's ability to quickly deploy a website hosting any type of data, user-friendly GUI, several programmatic interfaces and support for third party tools has proved to be an attractive solution for data managers who were in need of a rapid and reliable solution for their user community. BioMart has also proven to be a platform of choice for many smaller organizations that lack the necessary resources to embark on the development of their own data management solution. As a result, more and more database projects have become accessible through the BioMart interface. The arrival of these new resources coupled with the data federation technology provided by the BioMart software has galvanized the creation of the BioMart Community Portal. The federated model has proven to be very cost-effective since all development and maintenance of individual databases is left to the individual data providers. It also has proven to be very scalable as the internet and database traffic is handled by the local BioMart servers. As a result the BioMart Community Portal service has grown impressively not only in terms of available data but also the level of service. The BioMart community portal now averages over million requests per our services per day. Building on this level of service and the wealth of information that has become accessible through the BioMart interface, the BioMart Community Portal has effectively introduced a new, more scalable and much more cost-effective alternative to the large data stores maintained by specialized organizations.
